# Vertebral Column Pathology Diagnosis Using Ensemble Strategies Based on Supervised Machine Learning Techniques

**DOI:** 10.3390/healthcare12131324

**Published:** 2024-07-02

**Authors:** Alam Gabriel Rojas-López, Alejandro Rodríguez-Molina, Abril Valeria Uriarte-Arcia, Miguel Gabriel Villarreal-Cervantes

**Affiliations:** 1 Optimal Mechatronic Design Laboratory, Postgraduate Department, Instituto Politécnico Nacional—Centro de Innovación y Desarrollo Tecnológico en Cómputo, Mexico City 07700, Mexico; arojasl2101@alumno.ipn.mx (A.G.R.-L.); auriartea@ipn.mx (A.V.U.-A.); 2 Colegio de Ciencia y Tecnología, Universidad Autónoma de la Ciudad de México, Mexico City 06720, Mexico

**Keywords:** vertebral column disease, artificial intelligence, ensembled classifiers, pattern recognition, supervised learning techniques

## Abstract

One expanding area of bioinformatics is medical diagnosis through the categorization of biomedical characteristics. Automatic medical strategies to boost the diagnostic through machine learning (ML) methods are challenging. They require a formal examination of their performance to identify the best conditions that enhance the ML method. This work proposes variants of the Voting and Stacking (VC and SC) ensemble strategies based on diverse auto-tuning supervised machine learning techniques to increase the efficacy of traditional baseline classifiers for the automatic diagnosis of vertebral column orthopedic illnesses. The ensemble strategies are created by first combining a complete set of auto-tuned baseline classifiers based on different processes, such as geometric, probabilistic, logic, and optimization. Next, the three most promising classifiers are selected among k-Nearest Neighbors (kNN), Naïve Bayes (NB), Logistic Regression (LR), Linear Discriminant Analysis (LDA), Quadratic Discriminant Analysis (QDA), Support Vector Machine (SVM), Artificial Neural Networks (ANN), and Decision Tree (DT). The grid-search K-Fold cross-validation strategy is applied to auto-tune the baseline classifier hyperparameters. The performances of the proposed ensemble strategies are independently compared with the auto-tuned baseline classifiers. A concise analysis evaluates accuracy, precision, recall, F1-score, and ROC-ACU metrics. The analysis also examines the misclassified disease elements to find the most and least reliable classifiers for this specific medical problem. The results show that the VC ensemble strategy provides an improvement comparable to that of the best baseline classifier (the kNN). Meanwhile, when all baseline classifiers are included in the SC ensemble, this strategy surpasses 95% in all the evaluated metrics, standing out as the most suitable option for classifying vertebral column diseases.

## 1. Introduction

Process automation has been one of the main goals of research efforts since the third Industrial Revolution [1]. The advance of technological resources has impacted various sectors, including the visual [2], musical [3], chemical [4], and biological [5] sciences, besides the automation of mechanical processes [6]. One of the most significant research areas nowadays is computational science, where combining mathematics, logic, and electronics has led to the development of more powerful computational methods and techniques [7]. In recent decades, the popularity of pattern recognition techniques has significantly increased because of their wide range of applications regarding classification, data recovery, regression, and clustering problems [8,9].

Particularly, pattern recognition techniques have become incredibly reliable in classification applications due to their simplicity and well-established execution process, which helps solve problems where the correct data classification is crucial. Such an execution process of classifiers is summarized in the following steps [10]:

1. The first step is to create a desired information dataset. To accomplish this, it is necessary to consider the different outputs (classes) into which the information will be divided. Also, it is essential to determine the inputs and characteristics (attributes) from which the classes are to be evaluated [11].
2. Once the information is gathered, it must be analyzed and filtered. This step is essential for assessing the adequacy of the dataset in terms of the number of samples, the representativeness of the characteristics, and the accuracy of the element values, among other factors [12].
3. After determining the reliability of the dataset for analysis, a specific pattern recognition approach can be trained or directly executed, depending on its behavior, to obtain the optimal parameters (hyperparameters). These hyperparameters will categorize future data based on the chosen attributes [13,14].
4. In the last stage, a validation step is necessary, wherein the effectiveness of the classifier is assessed using new data (the test set) by evaluating performance metrics such as accuracy, recall, or precision [15,16].

The classification techniques based on pattern recognition provide the advantage of being easily applicable to data from any field without being limited to engineering approaches. This makes them valuable in contexts where the information is complex to depict or indecipherable to other disciplines [17]. An example of these applications is presented in [18], where an Artificial Neural Network (ANN) is utilized to classify catchments and their drivers based on seasonal water quality using spatio-temporal data. Another element may be found in the study conducted in [19], which aimed to categorize tea based on its harvest season using FT-IR fingerprinting. This target was achieved using the Principal Component Analysis (PCA) technique. These works explore the geographical and climatic factors as attributes that can enhance the engineering and production processes despite the limited knowledge about such topics among the individuals involved in the research.

Likewise, several works belong to highly specialized domains such as health, medicine, and biology, whose intricate possesses require expert analysis for accurate description and interpretation. However, pattern recognition classifiers continue to yield favorable results when applied to these circumstances. An element of this may be found in [20], where the researchers employed the k-Nearest Neighbors (kNN) classifier to determine the best control input by analyzing the surface electromyogram (sEMG) signals. Furthermore, particular works employ intricate and resilient adaptations of an initial pattern recognition technique, as demonstrated in [21]. That work utilizes a Convolutional Neural Network (CNN), an ANN variant, to categorize hypospadias. A comparative analysis was conducted between the CNN and fifty-three specialists, yielding comparable levels of accuracy at 90%.

Moreover, in certain elements, the classifiers utilizing pattern recognition approaches have achieved such a high level of trustworthiness that they are employed to enrich medical procedures. In [22], a Naïve Bayes (NB) classifier was employed to enhance cancer patients’ management and medical care after radiotherapy treatment. The classifier was employed to classify those patients based on their probability of relapse or progression.

However, although there are many different pattern recognition techniques, none will likely obtain perfect results on all existing data sets [23]. In light of this, it is advisable and essential to compare several pattern recognition techniques while implementing a classifier. This allows for selecting the most effective strategy, leading to notable enhancements. It is important to note that the performance of a classification approach can be significantly altered by the selection of different hyperparameters, even when using just one technique. Studies related to hyperparameter selection can be seen in [24,25,26,27]. Therefore, when aiming for classification, it is recommended to evaluate several classifiers in their basic form [28] and fine-tune their hyperparameters [29]. Considering this, the study described in [30] not only includes the implementation of classifiers such as ANN and random forest but also conducts comparisons to determine which classifiers are more effective for a given problem. Such work involves analyzing driver injury trends from a multi-class perspective. However, the study does not contain additional classification techniques for comparison. In [31], a study is conducted to compare several pattern recognition techniques, including kNN, ANN (with radial basis function), and Support Vector Machine (SVM). Those methodologies were utilized to categorize diseases of the vertebral column. Although the study includes many tests and validation criteria, it does not modify the hyperparameters of the classifier to evaluate its performance, nor does it experiment with other classifiers.

In addition, a preliminary classification comparison provides a more reliable selection of classifiers for practical or real-world applications and future research baseline comparing elements. Recently, the study conducted in [32] showed the superiority of the kNN classifier in automatic text classification compared to other machine learning techniques. As a result, the kNN classifier was chosen for further investigation. This kind of practice has become a common trend in medical applications. An example of this is presented in research like [33,34,35], where many classifiers were examined for the categorization of different critical diseases, and the winners in each study were distinct ANN architectures. In those cases, selecting the most effective classifier is crucial because the application is related to difficulties associated with medical treatments and patients’ quality of life. Likewise, in [36], a comparative analysis of many studies that employ pattern recognition approaches to classify diseases is carried out. The study showed that the Support Vector Machine (SVM) was the most widely used classifier among the studies. However, the Random Forest (RF) classifier achieved the highest level of accuracy. Similarly, in [37], different classifiers were empirically tuned, resulting in the kNN classifier as the most suitable option for categorizing vertebral column disease.

Nonetheless, it is essential to keep in mind that even if these pattern recognition techniques are based on a statistical approach, their solution relies on different process properties like geometrical, probabilistic, logical, etc. This means that each classifier takes advantage of different properties of the dataset, like its elements distribution or the attributes’ correlations. In recent years, an outstanding practice in the biology field for classification problems has been to use ensemble strategies to enhance the results of the classification problem beyond the limits of using a baseline classifier. An example of this is presented in [38], where an ensemble of three different classifiers (ANN-based) enhances the classification task of chromosomes, phages, and plasmids, which was a complicated endeavor for the baseline classifiers. Similarly, in [39], an RF ensemble (ensemble of decision trees) enhances COVID-19 case detection, surpassing the accuracy percentage limit obtained by DT and SVM baseline classifiers. However, it is important to note that in such works, the same baseline classifiers were used for their respective ensemble strategies, which limits the classification process to the characteristics of the baseline classifiers. Nonetheless, some works implement diverse baseline classifiers to take advantage of its properties, like in [40] where the kNN, SVM, and DT classifiers were ensembled through voting strategy to enhance the diagnosis and prediction of coronary artery disease with reduced feature subset. However, in [40], there is no mention of other ensemble strategies or the criteria to limit the ensemble strategy to those classifiers. Similarly, in [41], the voting strategy is used to classify cardiovascular disease through the ensemble of stochastic gradient descent, logistic regression, and support vector machine classifiers. Nonetheless, adding other classifiers tested in the same work is not considered.

Only a few works related to ensemble strategies can be found in the literature, particularly regarding the vertebral column illness classification problems (case of interest). In [42], different variants of SVM-based ensemble strategies are applied to different datasets, including vertebral column disease classification. Despite the SVM-based ensemble strategies achieving outstanding results in other classification problems with accuracies beyond 90%, the SVM-based ensemble strategies only achieved accuracies of around 85% in the classification of vertebral column disease. This outcome shows that ensemble strategies based on only one kind of baseline classifier process cannot be sufficient for all kinds of problems. Another example of this single-technique-based approach is presented in [43], where ensemble variations of the original random forest ensemble strategies are tested in the vertebral column illness classification, where none of the implemented proposals could surpass an accuracy of 85%, being limited by the process of only one baseline classifier. Another similar case is presented in [44], where a voting ensemble based on ANN variants is applied to vertebral column illness classification. Even if the proposal reached an accuracy of up to 90%, the ensemble is still limited to the ANN variants and their complexity.

Considering that different classifiers are based on different processes like geometrical, probabilistic, or logic [45,46], combining them can offer a major diversity to the solution of the ensemble strategy for the classification problem since the drawbacks of a particular baseline classifier can be compensated through the perks of another classifier. So, to the authors’ best knowledge, ensemble strategies mixing baseline classifiers, whose procedures are based on different approaches, have not been implemented in vertebral column disease classification. This forms the first contribution of this paper.

On the other hand, this study aims to analyze and contrast the performance of voting and stacking ensemble strategies based on auto-tuned supervised machine learning techniques in vertebral column orthopedic illnesses to provide insight into their characteristics and limitations with respect to metrics like accuracy, precision, recall, and F1-score, as well as evaluating the receiver operating characteristic curve. This study can help researchers choose appropriate pattern recognition approaches for future studies on problems with similar characteristics. Researchers and people in the area might use the presented information as guidelines to select an initial set of classifiers for vertebral column disease, determine which classifiers are the most useful in the ensemble to increase classification performance, and understand the ensemble behavior of popular ensemble strategies. The latter forms the second contribution of the paper.

The rest of the present work is distributed as follows: Section 2 describes, analyzes, and explains the preprocessing approach used in the vertebral column dataset employed in this work. Section 3 provides an operation process explanation of the implemented ensemble strategies and the baseline classifier that will shape the ensembles. Section 4 incorporates the analysis methodology, discusses the obtained results separately, and provides a general discussion at the end. Finally, Section 5 provides a detailed account of the conclusions drawn from this study and outlines potential areas for further research.

## 2. Description and Analysis of the Dataset

The dataset is acquired from the Vertebral Column dataset (VCDS) available at the following link: https://archive.ics.uci.edu/ml/datasets/Vertebral+Column [47] (accessed on 1 January 2024). The information was gathered and refined by Dr. Henrique da Mota in the Group of Applied Research in Orthopaedics (GARO) at the Centre médico-chirurgical de réadaptation des Massues in Lyon, France. There are 310 elements within the dataset, and each of them is evaluated through the following six biomechanical attributes (a brief description of the attributes is included):

(i) Pelvic incidence (PI): This is the angle between a line perpendicular to the sacral plate at its center and a line connecting the same point to the center of the bicoxofemoral axis [48].
(ii) Pelvic tilt (PT): This is the angle estimated between two reference lines. The first is a vertical line to the center of the femoral head. The second goes from the center of the femoral head to the midpoint of the sacral endplate [49].
(iii) Lumbar lordosis angle (LLA): The angle measured in the sagittal plane between the two ends of the lumbar curve [50].
(iv) Sacral slope (SS): The angle produced by a line parallel to the sacral end plate and a horizontal reference line [49].
(v) Pelvic radius (PR): The distance from the hip axis to the posterior–superior corner of the S1 endplate [51].
(vi) Grade of spondylolisthesis (GS): The grades are considered as follows: grade I represents 0–25%, grade II 25–50%, grade III 50–75%, and grade IV 75–100%. These percentages represent how much the cephalad vertebra has slipped anteriorly, related to the caudal vertebra. The superior endplate of the caudal vertebral body is divided into four equivalent quadrants, and the magnitude of the slip is based on the percentage of endplate uncovered as a result of the slip [52].

The dataset can be utilized for two classification problems. The first problem splits the set into three distinct categories: Normal (consisting of 100 elements), Disk Hernia (consisting of 60 elements), and Spondylolisthesis (consisting of 150 elements). The second problem scheme is more straightforward, as it just categorizes cases into two groups: Normal, which consists of 100 elements, and Abnormal, which consists of 210 elements. This study will concentrate on the initial option, which involves three distinct categories, to demonstrate the effectiveness of classification methods in achieving a more comprehensive and detailed categorization.

Before commencing the application of the classifiers, it is crucial to verify the usability of the dataset. In this particular version, the dataset does not exclude any information in the elements. However, the data exhibits an imbalance between the categories. This can be verified by computing the Imbalance Ratio IR (1).

(1) $$IR=\frac{\mathrm{Majority}\;\mathrm{class}\;\mathrm{elements}}{\mathrm{Minority}\;\mathrm{class}\;\mathrm{elements}}$$

According to the theory, a dataset is considered imbalanced if IR≥1.5 [53]. Thus, considering this in (3), it is confirmed that the dataset is imbalanced. As a matter of completeness, the relationship between the majority class and the second minority class (Normal class) is also estimated in (2). This last procedure is carried out only to show the great difference between the elements of the classes.

(2) $$IR_{2}=\frac{\mathrm{Spondylolisthesis}\;\mathrm{elements}}{\mathrm{Disk}\;\mathrm{Hernia}\;\mathrm{elements}}=2.5$$

(3) $$IR_{1}=\frac{\mathrm{Spondylolisthesis}\;\mathrm{elements}}{\mathrm{Normal}\;\mathrm{elements}}=1.5$$

**Figure 1:**
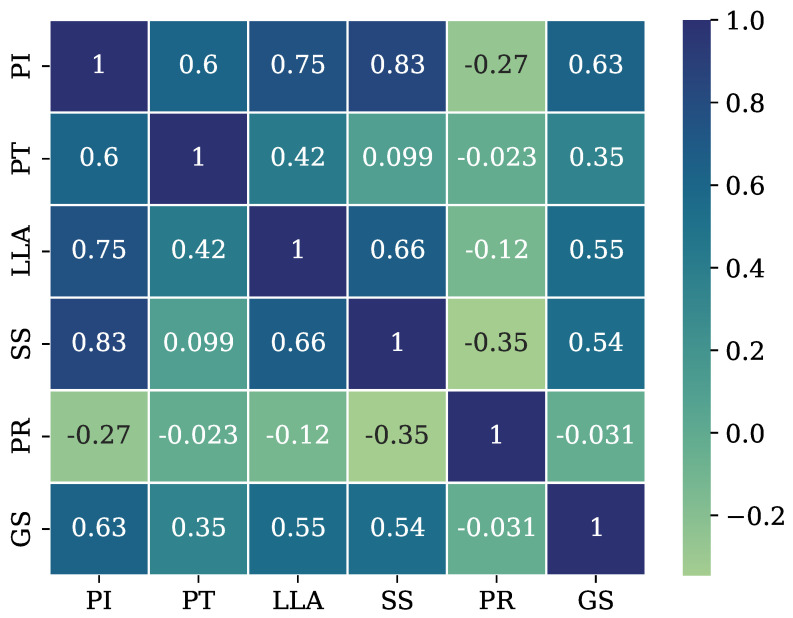
Heat map of the attributes’ correlation for the VCDS.

This dataset imbalance can decrease the classifiers’ potential due to insufficient elements for a representative and fair classification. So, the dataset is corrected through the Adaptive Synthetic Sampling Approach for Imbalanced Learning (ADASYN), which randomly creates additional samples for the minority classes [54,55] (oversampling correction strategy). The technique is employed to generate synthetic samples to achieve 150 elements for each class. This technique is used over other popular approaches, such as the Synthetic Minority Oversampling Technique (SMOTE), since ADASYN can create samples with more defined decision boundaries and is less susceptible to the noise produced by outliers [56]. Figure 1 displays the heat map illustrating the correlation among the attributes of the oversampled dataset. The depicted matrix displays the correlations between the attributes in the row labels and attributes in the column headers. A negative cell value indicates a negative correlation (inverse relationship), while a positive value expresses a positive correlation (direct relationship). A lower magnitude value in a cell represents independence between the correlated attributes, and a greater magnitude value means dependency between them. It is observed that the PR attribute has the lowest correlation magnitudes (the more independent attribute), while the PI attribute has the greatest correlation magnitudes (the more dependent attribute). Typically, strategies to boost the performance of a particular classifier (depending on the attributes’ correlations) can be implemented to reduce the dataset size by eliminating an attribute to reduce the computational load [57]. Nevertheless, given the limited size of this dataset, there may not be an enhancement in the computational load, and the performance can be affected [58]. Therefore, all the attributes are to be employed in the classification processes.

**Figure 2:**
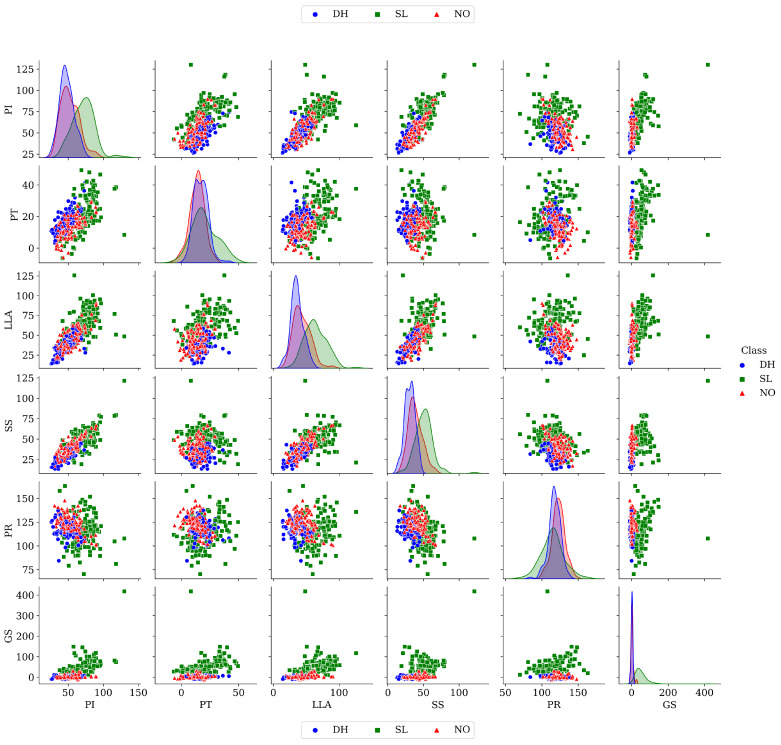
Pairwise principal component analysis of the VCDS.

Looking for a complete analysis of the employed dataset, the principal component analysis (PCA) matrix in a pairwise comparison among the attributes is presented in Figure 2. The distribution of the elements according to just one attribute is offered in the diagonal of this matrix, where the distributions of the DH, SL, and NO classes are depicted in blue, green, and red, respectively. It is observed that, for elements of the SL class, the attributes PI, LLA, SS, and GS have important differences against those of the other two classes (DH and NO). On the other hand, only the attributes LLA, SS, and PR have slight differences in the distributions of the DH and NO classes. Furthermore, the rest of the matrix of pairwise comparisons provided in Figure 2 shows the distribution of scattered elements of the three classes in each plane created between the considered attributes. The DH, SL, and NO classes are depicted in blue circles, green squares, and red triangles, respectively. Within these planes, it is observed that a high correlation exists between the elements of each class since the elements of the classes overlap. Moreover, the scattered elements of all the classes tend towards a linear behavior in those planes that use one of their axis components, the GS and PI attributes. This information could prove valuable in determining the key attributes to consider.

## 3. Classifier Methods

Since the dataset is already labeled, the classification methodology belongs to supervised learning [59]. As previously mentioned, this work aims to test different baseline classifiers whose methodologies may rely on diverse properties like geometrical, probabilistic/statistical, logical, or optimized learning [29,60]; these differences will enhance posterior ensemble strategies. Hence, the following baseline classifiers are included in the study:

### 3.1. Basic Classifier Methods

#### 3.1.1. k-Nearest Neighbors (kNN)

This strategy relies upon geometrical approaches considering the closeness of elements within a dataset. The approach takes into account the categories of the *k* nearest elements. So, kNN involves computing the distance $d_{i}$ between a new element to be classified $x^{\prime }$ and the elements x in the dataset (Table 1 provides commonly used distance functions [61,62]). Once the *k* nearest elements $\left\{x_{1},\cdots ,x_{k}\right\}$ have been identified, the following task is to determine the prevailing class among them. The main benefit of this approach is its lack of a training phase, which results in an immediate classification process. However, this strategy encounters the following issues:

1. It is necessary to find the correct *k* value, i.e., the number of elements to be considered in the distance comparison.
2. If a new element to be classified is situated in an area where the *k*-neighbors are evenly distributed among two or more classes, the classification is impossible (without using other additional implementations) because there is no dominant class.
3. If the attributes of the elements are discrete (categorical), the distances cannot be measured without additional valuations regarding the previous knowledge of the dataset.

#### 3.1.2. Naïve Bayes (NB)

This methodology utilizes a probabilistic approach to determine the possibility of an event (class) to occur by evaluating the attributes of a new element [63]. The calculation takes into account the initial probability of a *k*-th class, denoted as $P(c_{k})$, as well as the verisimilitude probability of an attribute $x_{i}$ given class *k*, denoted as $P(x_{i}|c_{k})$. Therefore, when a new element $x^{\prime }$ is classified, it computes the posterior probability using Equation (4). It is worth pointing out that, similarly to the previous technique, this one does not require a training phase but additional computational operations. This approach likewise offers the benefit of rapid application, and in addition to the previous one, it is capable of handling both discrete and continuous numbers. An inherent limitation of this strategy is its assumption of independence (low correlation magnitude) between the attributes of the elements within the dataset.

(4) $$P\left(c_{k}|\left\{x_{1},\cdots ,x_{n}\right\}\right)=\frac{P\left(\left\{x_{1},\cdots ,x_{n}\right\}|c_{k}\right)P\left(c_{k}\right)}{P(\left\{x_{1},\cdots ,x_{n}\right\})}=P\left(c_{k}\right)\prod_{i=1}^{n}P\left(x_{i}|c_{k}\right)$$

#### 3.1.3. Logistic Regression (LR)

**Figure 3:**
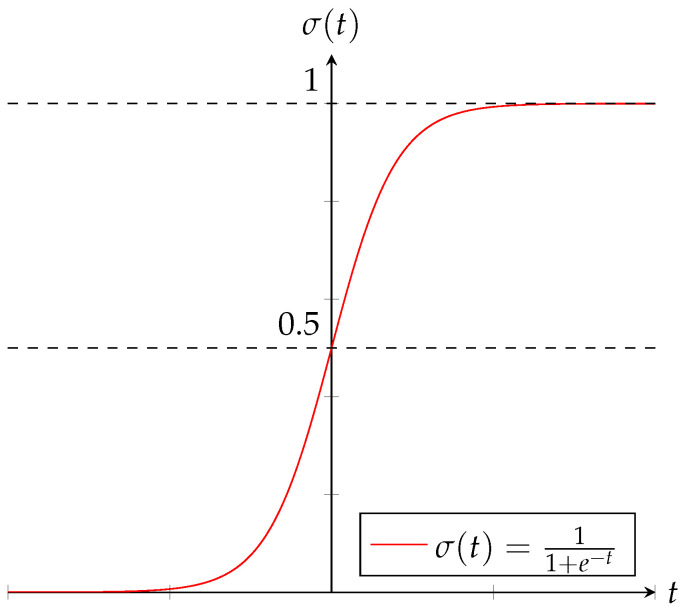
Sigmoid function example.

Despite its name suggesting a regression process, this technique is used as a classifier. The LR classifier implements a similar process to linear regression, where there is a weighted sum of the attributes plus a bias term. The vector form of the operation is defined as $x^{T}\theta$, where $x={\left[1,x_{1},\cdots ,x_{nx}\right]}^{T}$ is an element of the dataset and $\theta ={\left[\theta_{0},\cdots ,\theta_{nx}\right]}^{T}$ is the vector that includes the bias value and the weights of each attribute. The manner that LR performs classification tasks is through the implementation of a logical binary state function (5), where $\hat{p}=\sigma \left(x^{T}\theta \right)$, σ(·) is a Sigmoid function (see Figure 3) and $\hat{y}$ is the binary state of probability of an element to be included within a class [64]. Since the logistic functions mainly rely on the values of the bias and weights parameters, an optimization problem is solved in a training stage by minimizing a logistic regression cost function (6).

(5) $$\hat{y}=\left\{\begin{array}{cc} 0, & \mathrm{if}\;\hat{p}<0.5\\ 1, & \mathrm{if}\;\hat{p}\geq 0.5 \end{array}\right.$$

(6) $$J\left(\theta \right)=-\frac{1}{m}\sum_{i=1}^{m}\left[y^{\left(i\right)}\log\left({\hat{p}}^{\left(i\right)}\right)+\left(1-y^{\left(i\right)}\right)\log\left(1-{\hat{p}}^{\left(i\right)}\right)\right]$$

Different optimization algorithms (solvers) can be selected to solve such problems, where the next approaches are most common:

- Gradient-based:
  – Library for Large Linear (liblinear).
  – Stochastic Average Gradient (sag).
  – Stochastic Average Gradient Accelerated (saga).
- Hessian-based:
  – Limited-memory Broyden–Fletcher–Goldfarb–Shanno (lbfgs).
  – Newton–Cholesky algorithm (newton-cholesky).
  – Newton conjugated gradient (newton-cg).

#### 3.1.4. Linear and Quadratic Discriminant Analysis (LDA and QDA)

Similarly to the NB classifier, the discriminant analysis method relies on the Bayes theorem. The discriminant analysis process computes a regression function that splits the dataset elements into the evaluated classes. The regression function is a posterior probability density function as in (7), which represents the probability of a new element $x\in R^{d}$ to be classified as the *k*-th class. The constant *d* represents the number of attributes of each element, and $\Sigma_{k}\in R^{d\times d}$ is the covariance matrix of the *k*-th class [65]. The main difference between LDA and QDA is that the first one considers that each class can be represented by separated Gaussian distributions where all these distributions (classes) share the same covariance matrix. On the other hand, the QDA classifier considers that each distribution possesses an independent covariance matrix. A particular case of the QDA classifier employs diagonal covariance variance matrices (where the attributes are independent between them), making the QDA classifier the same as the NB classifier. Since these classifiers are also based on a probabilistic strategy, they require independence among the attributes (low correlation magnitude). If the attributes are not independent, there will be no statistical significance to complete the classification successfully.

(7) $$P\left(x|y=k\right)=\frac{1}{{\left(2\pi \right)}^{\frac{d}{2}}{\left|\Sigma_{k}\right|}^{\frac{1}{2}}}\exp\left(-\frac{1}{2}{\left(x-\mu_{k}\right)}^{T}\Sigma_{k}^{-1}\left(x-\mu_{k}\right)\right)$$

Finally, a piece of noteworthy information is that the LDA classifier can select its solver to compute the eigenvector and eigenvalues used for the function regression [66]. Next, a brief description of popular solvers is presented:

- Single value decomposition (*svd*): This approach decomposes a matrix into three matrixes, helping to reduce dimensionality, which aids attribute extraction tasks.
- Least Squares solution by QR decomposition (*lsqr*): Iterative method that approximates the eigenvector and eigenvalues used in the function regression. It is helpful to reduce the computational burden in large dataset classification problems.
- Eigenvalue decomposition (*eigen*): This approach directly computes the exact eigenvalue decomposition of the covariance matrix. This approach is the most computationally intensive but achieves an exact solution.

#### 3.1.5. Support Vector Machine (SVM)

**Figure 4:**
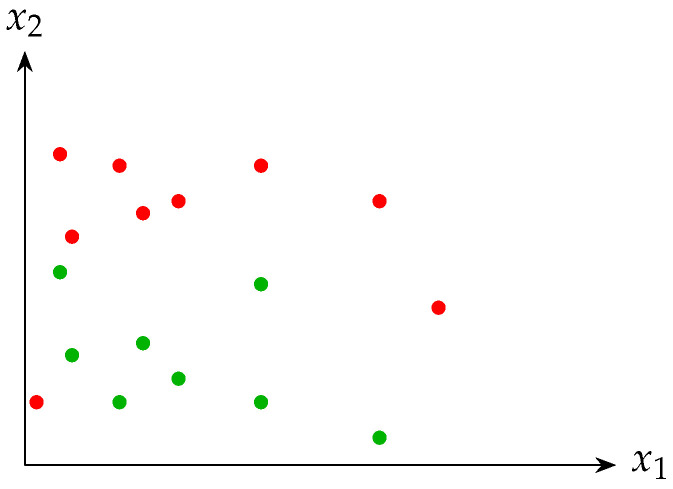
Example of SVM classifier with linear kernel.

This geometrical approach seeks to divide the elements of a dataset based on their classes using a hyperplane (or support vector) [64]. Figure 4 illustrates an example of the separation of two distinct classes (shown by red and green dots) by a dashed line (hyperplane). The division seeks to minimize the distance between the hyperplane and all elements of a given class. The equation of the hyperplane is determined by a kernel function $k(x,x^{\prime })$ [67]. Table 2 provides a list of commonly used kernel functions that will be implemented in this study. This method is renowned for its high level of accuracy, as it incorporates an optimization problem that considers all elements throughout the training stage. Yet, this attribute also results in an elevated computational time. An inherent limitation of this method arises when it is used on datasets where the elements possess attribute values that are overlapped. Nevertheless, this limitation can be mitigated by increasing the number of hyperplanes used in the calculation despite the resulting increment in the computational burden.

#### 3.1.6. Artificial Neural Network (ANN)

**Figure 5:**
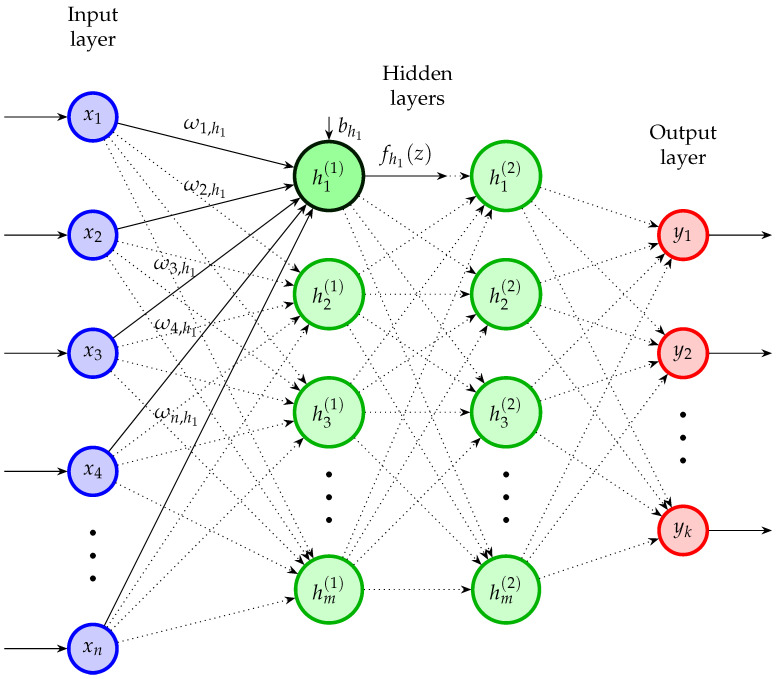
Artificial Neural Network diagram.

This classifier utilizes a concatenation of individual neurons, resembling the network depicted in Figure 5. Notably, the ANN architecture employed in this paper is a feed-forward topology with hidden layers. Every neuron adheres to two fundamental processes [68]. The first hidden layer’s first neuron $h_{1}^{\left(1\right)}$ is analyzed to present an example. The first process is to solve (8) by computing the sum of the products of each input $x_{i}$ with its corresponding weight $\omega_{i,h_{1}}$ that are connected to the neuron $h_{1}^{\left(1\right)}$, and then adding the bias $b_{h_{1}}$ of the neuron. Subsequently, the outcome $z_{h_{1}}$ from the preceding phase is employed in an activation function $f_{h_{1}}\left(z_{h_{1}}\right)$ (Table 3 displays popular activation functions), with the resulting output serving as the input for the subsequent neurons in the next hidden layer or as the ultimate result in the output layer. All the neurons of the network execute these two processes. At the training stage, an optimization problem is formulated to determine the optimal weights and biases of the neurons to enhance the neural network performance. The use of an optimization problem in the ANN makes it one of the most precise and dependable classifiers. However, it is essential to note that the training step is somewhat slower than alternative approaches. Additionally, it is crucial to note that the ANN approach relies on other hyperparameters like the learning rate (step size of the optimization algorithm) and the dimension/structure of the hidden layers. Another crucial factor affecting an ANN’s behavior is related to its solver algorithm (optimization algorithm), which computes the ANN’s weights and biases. Some popular optimization algorithms are the stochastic gradient descent (sgd), lbfgs, and the adaptive moment estimation (adam) algorithms.

(8) $$z_{h_{1}}=\sum_{i=1}^{n}\omega_{i,h_{1}}x_{i}+b_{h_{1}}$$

#### 3.1.7. Decision Tree (DT)

**Figure 6:**
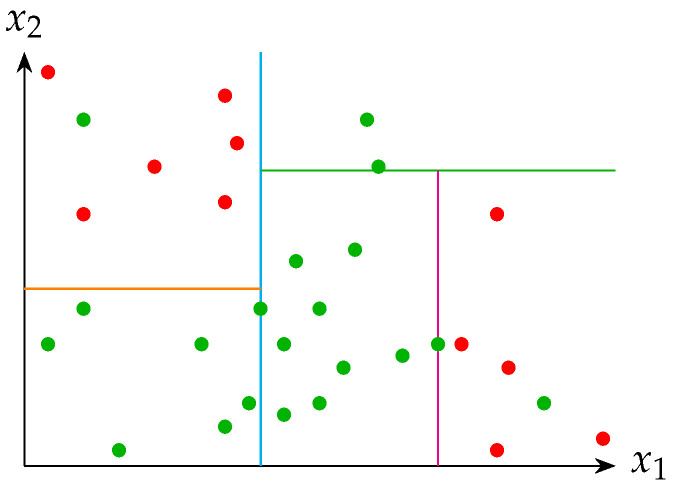
Example of DT classifier for two attributes evaluation.

In contrast to the previous methods, this strategy relies on logical reasoning rather than a mathematical foundation. Visually, the DT method can bear a resemblance to the SVM approach. However, instead of employing a hyperplane, DT utilizes orthogonal divisions based on the datasets’ attributes [69]. Figure 6 depicts a fundamental illustration of how the method divides the elements of two classes (red dots and green dots). The elements possess two attributes, denoted as $x_{1}$ and $x_{2}$. This method aims to identify the values (within the range of the attributes) that divide the elements into two groups, each consisting of components belonging to just one class. Supposing the new groups fail to meet the specified criteria, the procedure will be iterated, creating a hierarchical structure of conditional functions known as a *Tree*. This approach relies upon several elements, including

- The criterion to split the tree into a new branch. Some of the most common criteria are [70]
  – Gini: This measures how often a randomly chosen element from a set would be incorrectly labeled if it was randomly labeled according to the distribution of labels in a subset.
  – Entropy: This measures the randomness in the dataset split.
  – Logarithmic loss (log_loss): This measures the performance of a classification model where the predicted output is a probability value between 0 and 1.
- The minimum number of elements required to halt the divisions.
- The number of splits based on certain criteria.
- The number of layers in the tree.

Nevertheless, when appropriately calibrated, the classifier emerges as one of the most precise techniques due to its ability to classify datasets containing cases with non-continuous attribute ranges. However, this method has limitations when it comes to the various hyperparameters that need to be adjusted, including the criterion (Gini or entropy for measuring impurity), splitter (random or best for selecting the split point on each node), samples split (minimum number of elements required to split a node), and sample leaf (minimum number of elements needed to convert a node into a leaf/class).

### 3.2. Ensembled Classifier strategies

**Figure 7:**
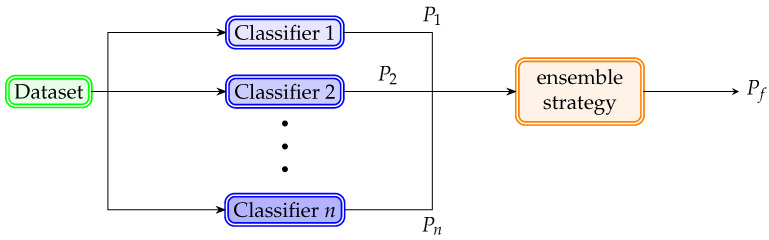
Ensemble classifier scheme.

Since the individual classifiers work through different approaches, their results may vary in their accuracy [71]. In some practices, a classifier might better classify particular elements, while another classifier might be more accurate with different ones. Therefore, a strategy to enhance the classification process is to ensemble multiple pattern recognition techniques [72,73]. Figure 7 displays an example of *n* classifiers gathered to find a final forecast $P_{f}$ by evaluating their *n* individual predictions through an ensemble strategy.

Next, brief descriptions of the most notable ensemble strategies are presented:

- Voting classifier (VC) [74]: As its name suggests, this strategy performs a voting process with the baseline classifiers implemented, where the final forecast $P_{f}$ is the majority of the individual predictions $\left\{P_{1},\cdots ,P_{n}\right\}$. A variant of the voting process assigns weights to each of the individual predictions considering the classifiers’ reliability $\left\{w_{1}P_{1},\cdots ,w_{n}P_{n}\right\}$.
- Stacking classifier (SC) [75]: This ensemble strategy uses an additional classifier (meta-classifier) to compute the final prediction $P_{f}$. This meta-classifier is usually an LR classifier due to its simplicity by providing a smooth interpretation of the predictions made by the baseline classifiers.
- Random Forest (RF) [76]: This method is similar to the voting classifier, since the final prediction $P_{f}$ is made by selecting the majority of the individual predictions of the baseline classifiers. The most representative characteristic of the RF is that it only uses Decision Trees as baseline classifiers. Each of these baseline classifiers is different from each other. Another noteworthy point is that the baseline classifiers of the RF evaluate only particular attributes of the elements of the dataset. This characteristic makes the baseline classifiers less complex than a unique decision tree to perform the whole classification process.

## 4. Results

The classifiers used in this work are developed using the Python 3.12.1 programming language. The classification algorithms use the *scikit-learn* library [64,77]. It is important to note that the classifications were performed using a computer equipped with an Intel^®^ Core^TM^ i7-7700HQ CPU running at a clock speed of 2.80 GHz and 16 GB of RAM.

### 4.1. Experimentation Methodology

Before addressing the results, explaining the methodology employed to carry out the experiments is essential. Next, a brief description of the stages used is offered:

- Firstly, the hyperparameters of the baseline classifiers presented in this work are tuned through a grid-search K-Fold cross-validated strategy. This process aims to find the most suitable hyperparameters per classifier, hence the best version of the baseline classifiers. A thorough description of this process is presented in Section 4.2.
- An analysis of the results of the tuned baseline classifiers is presented in Section 4.3. This analysis aims to provide insight into the baseline classifiers and find the best for the particular problem of the vertebral column disease classification.
- Section 4.4 encompasses the description and analysis results of different ensemble strategies (with subvariant proposals). This analysis highlights the behavior and performance of the employed ensemble strategies when different baseline classifiers are employed, providing a better understanding of their advantages and limitations.
- Finally, a general discussion is presented in Section 4.5. This discussion not only highlights the results of the baseline classifiers and the ensemble strategies but also discusses their trustworthiness in this particular medical problem for vertebral column disease classification.

### 4.2. Baseline Classifiers’ Hyperparameter Tuning

The baseline classifiers are tuned through the grid-search strategy to find the most suitable hyperparameters for the particular problem of vertebral column disease classification. The tuning process employs accuracy as the scoring metric for these tests, which is a well-recognized and dependable metric in pattern recognition techniques [78,79]. Considering the dataset’s reduced size, the K-Fold Cross-Validation (KFCV) process with a value of K=10 is employed [80] to analyze the diverse characteristics of the dataset instead of training the classifier with limited information, and also to ensure a robust evaluation. In each classifier, the grid-search strategy uses the most popular classifier hyperparameters. Next, a brief description of the hyperparameter variations used per baseline classifier in the grid-search tuning process is offered. Also, it is worth pointing out that the NB and QDA classifiers do not require a hyperparameter training process since their classification process is directly performed through probabilistic approaches as presented in Section 3.1.2 and Section 3.1.4, respectively. Moreover, it is worth remarking that discrete hyperparameters as the distance metric, activation functions, optimization algorithm solvers, or kernels in this work are restricted to the most popular, open-access options, yet within the literature are other options that have achieved important results.

- kNN classifier: The grid-search process varies the distance metric between Euclidean and Manhattan. Also, the number of *k* neighbors is evaluated from 1 to 30, i.e., k∈{1,⋯30}, considering the maximum permissible of the K-Fold process.
- LR classifier: In this classifier, the optimization algorithm (solver) used to perform the training is selected among the liblinear, sag, saga, lbfgs, newton-cholesky, and newton-cg algorithms.
- LDA classifier: In this classifier, the solver employed to compute the eigenvector and eigenvalues used for the function regression is chosen among the *svd*, *lsqr*, and *eigen* strategies.
- SVM classifier: The grid-search strategy evaluates the kernel function employed to create the hyperplane to separate the dataset element varying between linear, polynomial, radial basis, and sigmoid functions. Regarding the case of a polynomial function kernel, its degree *d* varies between 2 and 10, i.e., d∈{2,⋯10}. This limit is considered through an empirical observation where higher polynomial degrees increased the computational burden without enhancing the classification task. Such characteristics of polynomial degree increment have been analyzed in [81] with similar outcomes.
- ANN classifier: The grid-search strategy evaluates multiple hyperparameters like the number of hidden layers (between 1 and 10) and the number of neurons in the hidden layers (between 1 and 10). Also, the activation function used in the neurons is among identity, logistic, tanh, and relu functions (see Table 3). Moreover, the optimization algorithm (solver) used in the grid-search strategy varies between lbfgs, sgd, and adam. Finally, the last hyperparameter tuned is the learning rate $l_{r}$ varying it between 0.001 and 0.9 with a step size of 0.005, i.e., $l_{r}\in \left\{0.005,0.01,\cdots ,0.895,0.9\right\}$. The range regarding learning rate, step size, number of hidden layers, and their number of neurons are established considering that the boundaries range set computational burden limits (regarding time) where other classifiers achieved acceptable outcomes without further endeavor.
- DT classifier: The grid search variates multiple hyperparameters like the criterion to split the elements of the dataset between gini, entropy, and log_los. Also, the splitter technique can be optimization-based (best) or stochastic-based (random). Moreover, the maximum depth of the tree varies between 5 and 10. Moreover, the minimum of samples to consider a node a leaf changes between 1 and 10. Finally, the minimum number of samples to split a node is between 1 and 10. Similarly, the hyperparameters tuned within ranges are selected considering boundaries where the computational burden limits are not surpassed.

Finally, Table 4 presents the baseline classifiers and their tuned hyperparameters that yield the highest reported accuracy.

### 4.3. Baseline Classifiers Results

Table 5 presents the results of the tuned baseline classifiers, where the first column depicts the evaluated classifier. The accuracy, precision, recall, and F1-score of the KFCV process obtained are presented from the second to the fifth columns, respectively. Looking for a complete analysis, the classifier’s confusion matrix is presented in the sixth column where the labels “DH”, “SL”, and “NO” stand for disk hernia, spondylolisthesis, and normal, respectively (which are the possible classes). The row label of the matrix indicates the actual classes, and the column label represents the class proportioned by the classifier. On each three-by-three matrix, the sum of each row equals the total of elements per class, i.e., DH, SL, and NO have 150 elements each. The number within the diagonal of each matrix indicates the correct classified elements (CCE) of the dataset (the CCE evaluation is presented in the seventh column of Table 5), and the rest of the cells are the misclassified elements.

Considering this is a medical problem where the misclassification of diseases might worsen the patient’s health, another crucial evaluation is related to the misclassified disease elements (MDE) valuation. The MDE valuation considers those elements of DH and SL classes misclassified as elements of the NO class. These errors are more important because they might cause the patients to remain with their illnesses instead of starting an accurate medical treatment. Other kinds of misclassification might lead to the start of medical treatment when the patient is healthy. These last misclassifications are not evaluated in this work because they might be corrected throughout the treatment (future medical analyses) by medical experts. The MDE evaluation of the baseline classifiers is presented in the eighth column of Table 5.

The last column of Table 5 includes the time metric, which considers the classifiers’ hyperparameter tuning, training, and implementation. It is essential to remember that each baseline classifier relies on different properties and requires different computational capabilities and resources. Hence, this last metric could be important for future implementation in real-world healthcare devices or future research considerations.

Furthermore, the best-obtained outcomes per evaluated metric in Table 5 are in boldface. Also, the classifiers are ranked according to their achieved CCE, where classifiers’ ranks are placed as a supper index in the classifier names of the first column. Finally, to achieve a straightforward understanding of the results, the following highlights are provided:

- The kNN classifier yields the most outstanding results, being the only one whose accuracy, precision, recall, and F1-scores are above 0.9. This is reflected in its CCE evaluation, where it correctly classified 427 elements. Also, the kNN classifier is the most trustworthy approach for this particular medical application since it only misclassified four disease elements as normal elements, i.e., MDE = 4.
- It is noteworthy that the SVM classifier achieves the second-best outcomes. This is interesting since both SVM and kNN classifiers use geometrical strategies. Nonetheless, it is important to mention that the SVM classifier drops its competitiveness against the kNN classifier by decreasing its CCE evaluation in 10.07%. The SVM’s MDE evaluation (MDE = 8) is twice greater (worse) than that reported by the best classifier.
- The DT classifier gets third place by reducing its CCE evaluation to 12.64% concerning the best classifier. However, even if its CCE evaluation is worse than the SVM classifier outcome, the DT classifier yields more reliable results since its MDE evaluation is lower (MDE = 6).
- The ANN classifier reaches fourth place, followed by the LR classifier, where both obtain similar outcomes. Interestingly, even if the ANN classifier is based on a more complex process, it does not obtain better results.
- The classifiers based on probabilistic strategies (NB, LDA, and QDE) report the worst outcomes. Their poor performance is related to the dataset characteristics, where there are high correlations among the attributes, as observed in Figure 1. This characteristic is against the operation principle of the probabilistic classifiers, which require statistical independence among the attributes evaluated.
- Particularly, regarding the MDE, it is observed that the baseline classifiers tend to misclassify the SL elements as NO elements. Notably, the SL elements were the majority class in the original imbalanced data.
- It is observed that the baseline classifier based on probabilistic strategies requires less time to be implemented, but their classification performance is lower against other classifiers. On the other hand, it is interesting that the best-baseline classifier (kNN) is 26.6 times faster than the second best-baseline classifier (SVM), indicating that the vertebral column disease classification task does not imply a computational waste endeavor.

**Figure 8:**
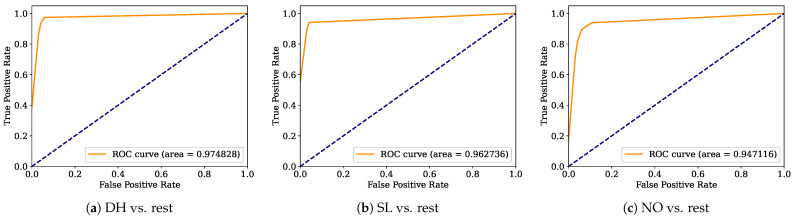
kNN ROC-AUC results.

**Figure 9:**
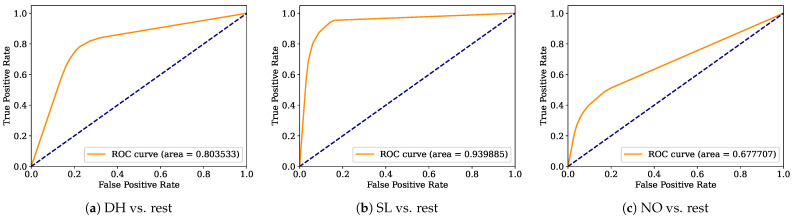
NB ROC-AUC results.

**Figure 10:**
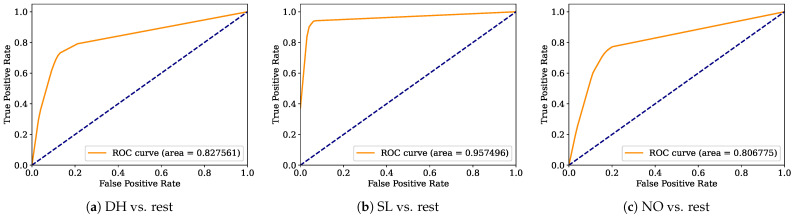
LR ROC-AUC results.

**Figure 11:**
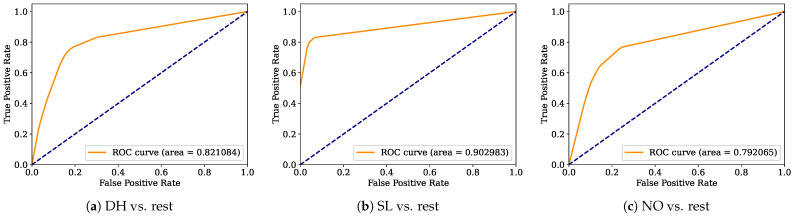
LDA ROC-AUC results.

**Figure 12:**
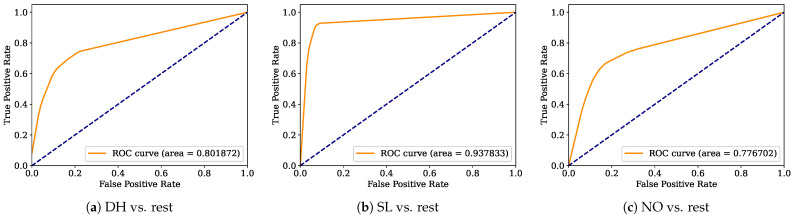
QDA ROC-AUC results.

**Figure 13:**
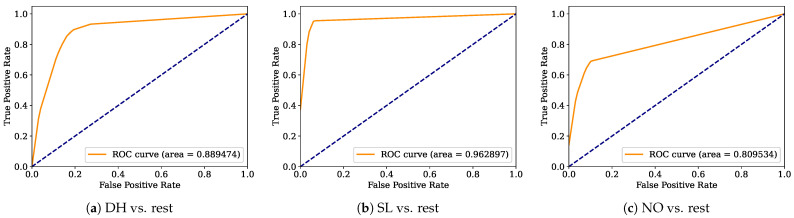
SVM ROC-AUC results.

**Figure 14:**
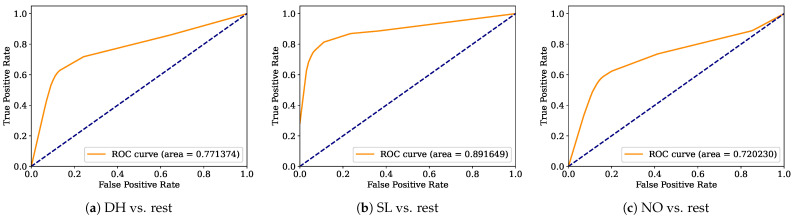
ANN ROC-AUC results.

**Figure 15:**
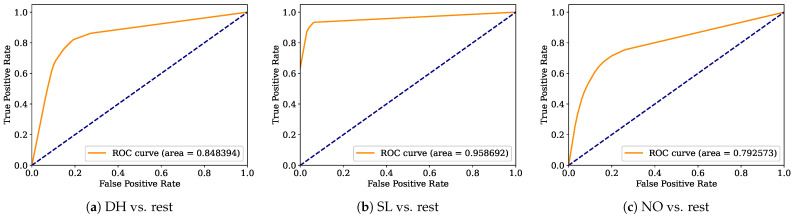
DT ROC-AUC results.

Aiming to provide a more robust analysis of the baseline classifiers, the Receiver Operating Characteristic curves (ROCs) and their area under the curve (ROC-AUC) are computed. Figure 8, Figure 9, Figure 10, Figure 11, Figure 12, Figure 13, Figure 14 and Figure 15 display the ROC-AUC results of the kNN, NB, LR, LDA, QDA, SVM, ANN, and DT baseline classifiers, in that order. Each graphic represents the relationship between the true-positive rate (y-axis) of an evaluated class against the false-positive rate (x-rate), where the ROC is depicted by a red line whose area represents the statistical power as a function of the Type I Error of the decision in a binary state (correctly classified or misclassified). Also, in these graphs, the dark blue-dashed line represents the probabilistic behavior of a random classifier (useless performance), where if the ROC curve is farther away from the random classifier, it implies that the evaluated classifier performs better for the assessed class. Otherwise, the classifier has low trustworthiness. Finally, in this set of graphs, all subfigures (a), (b), and (c) display the ROC-AUC of the assessed classes DH, SL, and NO against the rest of the classes, respectively.

The ROC-AUC outcomes corroborate the kNN as the best baseline classifier since it is the only one whose ROC-AUC results surpassed 0.9 in all the evaluated classes. Also, regarding the classification problem, it is observed that the easiest class to classify is the SL, where all the baseline classifiers (except for the kNN) achieved the best result in the ROC-AUC evaluation of this class. Also, it is observed that the DH and NO classes are more complicated to classify, confirming the information of the pairwise principal component analysis presented in Figure 2, where the distributions of each attribute per class show that the dataset’s elements of DH and SL classes present overlapping in all attributes. This characteristic limits the performance of the baseline classifier, particularly those that rely upon probabilistic approaches (NB, LDA, and QDA).

### 4.4. Ensembled Classifiers Results

Five different ensembled classifiers are executed to analyze the behavior of ensemble classifier strategies. The first one is the RF approach. The second and third methods are the voting and stacking classifiers, respectively, in a primary version, where the baseline classifiers used for their ensemble strategies are the top-three ranked classifiers according to Table 5 (hereinafter referred to as VC_top_ and SC_top_, respectively). Finally, the fourth and fifth methods to compare are also the voting and stacking classifiers, respectively, with the main difference being that they use all the baseline classifiers for their ensemble strategies (hereinafter referred to as VC_all_ and SC_all_, respectively). These last four methods consider no weighting among the included baseline classifiers, aiming to provide the same relevance to the individual results and excluding unknown preferences in the final ensemble results.

Table 6 presents the outcomes of the tested ensemble strategies, following a similar structure used to report the results of the baseline classifiers with the best-obtained outcomes per evaluated metric in boldface. The following noteworthy points are offered:

- Regarding the ensemble strategies that used all the baseline classifiers, it is observed that the SC_all_ ensemble yields the best outcomes, where the SC_all_ ensemble is the only ensemble strategy able to achieve results above 0.95 in all the evaluated metrics. These results make the SC_all_ ensemble the most outstanding option among all the ensembles and baseline classifiers. On the other hand, it is observed that the VC_all_ ensemble cannot boost its performance beyond the best baseline classifier (kNN).
- Concerning the ensemble strategies that use only the three best baseline classifiers, it is observed that the VC_top_ and SC_top_ ensembles have similar outcomes to the best baseline classifier (kNN). Particularly, the VC_top_ ensemble has the same kNN’s outcomes in all the evaluated metrics, and the SC_top_ ensemble drops kNN’s competitiveness by increasing its MDE valuation, misclassifying six disease elements as normal elements.
- The RF ensemble is the less promising ensemble strategy. Nonetheless, it is observed that the RF ensemble improves by 6.04% the CCE valuation of the DT classifier (its baseline classifier). These results show that an ensemble strategy that uses only one kind of baseline classifier has a limited performance, reaching only small improvements.
- Regarding the MDE metric, it is observed that, as with the baseline classifier, the ensemble strategies tend to miscategorize the SL elements as NO ones. This can result from the overlapping PT and GS dataset’s attributes, as observed in Figure 2. This kind of error might be mitigated by removing from the dataset the overlapped attributes, yet the best strategy is to consider additional attributes that clarify the differences between these classes. Therefore, in clinical problems, the machine learning results must be ratified by an expert in the field [82].
- Since the ensemble strategies work with already tuned baseline classifiers except for the RF ensemble strategy, the time measurement only considers the time required to complete the classification task, looking for a fair comparison. Particularly regarding the VC and SC ensemble strategies, it is clear that the variants that use all the baseline classifiers require more computational time than the variants that use only the top three, which is an expected result, according to related works like [83,84].

**Figure 16:**
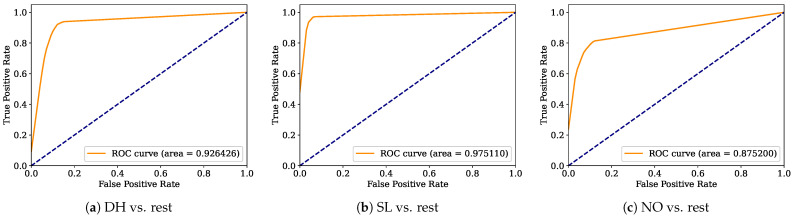
RF ROC-AUC results.

**Figure 17:**
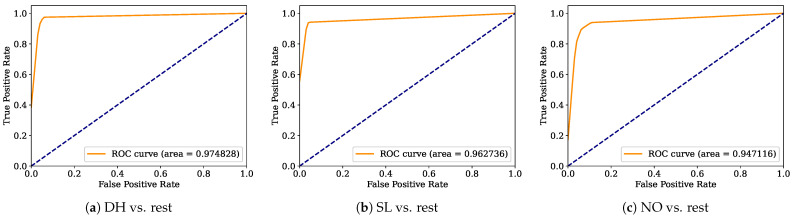
VC_top_ ROC-AUC results.

**Figure 18:**
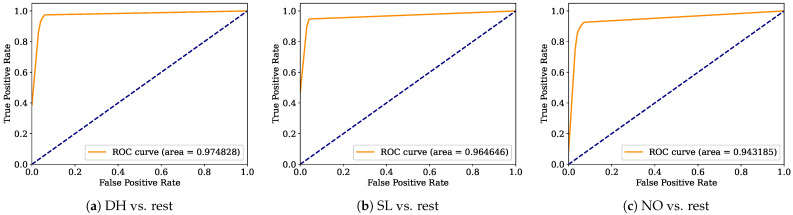
SC_top_ ROC-AUC results.

**Figure 19:**
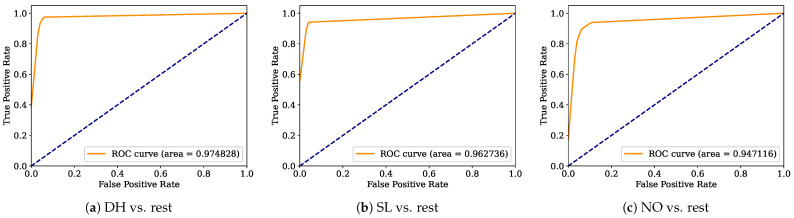
VC_all_ ROC-AUC results.

**Figure 20:**
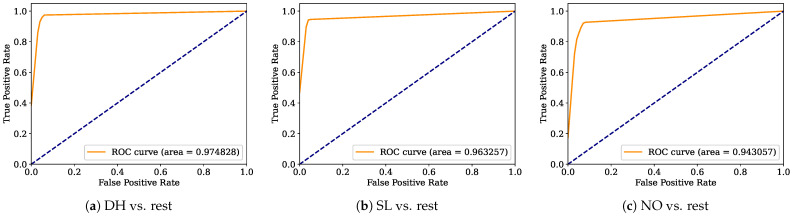
SC_all_ ROC-AUC results.

Looking for a deep analysis of the ensemble strategies, Figure 16, Figure 17, Figure 18, Figure 19 and Figure 20 display the ROC-AUC outcomes of RF, VC_top_, SC_top_, VC_all_, and SC_all_ ensemble strategies, respectively, following the same structure used in the ROC-AUC evaluation of the baseline classifiers. It is observed that the RF ensemble strategy is the worst ensemble strategy, since it is the only one unable to surpass 0.9 in the ROC-AUC of the NO class. Concerning the rest of the ensemble strategies, it is observed that all of them yield similar performance to the best-baseline classifier (kNN). Nonetheless, it is essential to remember that the SC_all_ ensemble strategy is the only one able to surpass 0.95 (kNN limit) in all the other evaluated metrics.

### 4.5. General Discussion

Considering the above-reported outcomes of baseline classifiers whose operation relies on different approaches, it is observed that the kNN yields the best performance among the baseline ones, presenting a significant difference of 10.0% (regarding the correctly classified elements) against the second-best (SVM). Also, it is observed that most of the classifiers present similar outcomes, obtaining values of accuracy, precision, recall, and F1-score in ranges from 0.81 to 0.85. Nonetheless, it is noteworthy that the ones whose operation relies on probabilistic approaches (NB, LDA, and QDA) are below those ranges, making them the least reliable for the problem of vertebral column disease classification. Moreover, an ROC-AUC analysis corroborates the kNN as the best baseline classifier since it is the only one that surpassed 0.9 in the curve areas related to the three possible classes.

Nevertheless, the ensemble strategies can outperform the results of the baseline classifiers. In particular, it is observed that the SC ensemble, when all the baseline classifiers are considered (SC_all_), is the only one that surpasses the accuracy, precision, recall, and F1-score beyond 0.95 (best-reported results among ensembles and baseline classifiers). On the other hand, when only the top-three baseline classifiers are used in the SC ensemble (SC_top_), the performance is limited to the best one (kNN). This shows that the more baseline classifiers are used in the SC ensemble strategy, the more representative input of the meta-classifier is created. On the other hand, the VC ensembles with the top-three and all baseline classifiers (VC_top_ and VC_all_, respectively) are limited to the best baseline classifier (kNN) performance. This is attributed to the significant difference between the kNN classifier and the rest of the baseline classifiers, making the rest of the baseline classifiers’ votes inconsequential. It is also observed that the RF ensemble is the less promising approach, which is attributed to the fact that all its baseline classifiers operate with the same principle, making the RF ensemble unable to face different conditions in the variance of the dataset. This is also confirmed through the ROC-AUC analysis, where the RF ensemble strategy cannot surpass the 0.9 curve area in the probability of classification related to the NO class, while the rest of the ensemble strategies reached similar outcomes to the best baseline classifier (kNN).

On the other hand, the use of ensemble strategies enhances the trustworthiness (reliability) of the classification process. This is observed in the decrement of misclassified elements (MDE), reflecting that fewer disease elements are misclassified as healthy ones (NO class), increasing the reliability of the ensemble strategies over the single baseline classifiers. An example of this can be observed through the second-best baseline classifier (SVM), which achieves the second-highest scores in the valuation of accuracy, recall, precision, and F1-score. Yet, its MDE is 100% greater (worse) than the best baseline classifier (kNN), resulting in an unreliable classification. In the case of the ensemble strategies, it is observed that in the worst of the cases (RF and SC_top_), the MDE results are only 50% greater, indicating that the ensemble strategies classification is more reliable than the baseline results. Moreover, it is observed that for some baseline classifiers (for instance, NB), the MDE valuation increases by 500% (the classification is worse), and when this baseline classifier is considered in the ensemble strategies (VC_all_ and SC_all_) the proposals provide more robust outcomes in the classification.

Finally, it is worth pointing out that this study is mainly limited to factors related to the available vertebral column dataset, the machine learning strategies implemented, and the resource consumption required for obtaining the ensemble results. The first limitation encompasses the imbalance ratio between the classes of the dataset and the oversampling technique used for the correction (ADASYN), which may overfit the dataset with synthetic instances that closely resemble the original data (in the case of noisy data, the class may increase mislabeled instances). Another limitation related to the dataset is the correlation between the attributes of the elements, which affected the misclassification of some elements, particularly those of the SL and NO classes. Also, this study is limited to some of the most popular, easy-access baseline classifiers, taking advantage of fundamental ML technique processes (geometric, probabilistic, logic, and optimization). Another limitation of this study is related to the resource consumption required for obtaining the ensemble results, which increases with respect to the baseline classifiers, where some ML processes imply higher computational loads.

## 5. Conclusions

Pathology diagnosis is one of the medical fields in which machine learning techniques help practitioners. Biomedical data classification is regarded as a delicate and challenging endeavor. This research examines VC and SC ensemble strategies using the three most promising auto-tuned baseline classifiers and a complete set of auto-tuned baseline classifiers evaluated for automatic diagnosis of vertebral column orthopedic illnesses. The comparative study with respect to the most popular pattern recognition techniques (kNN, NB, LR, LDA, QDA, SVM, ANN, and DT) indicates that using a meta-classifier in the SC ensemble strategy provides better outcomes when all the baseline classifiers are considered. In particular, the SC_all_ ensemble achieves values above 0.95 in evaluating the accuracy, recall, precision, and F1-score metrics. Nonetheless, it is noteworthy that when the SC ensemble strategy employs only the top-three baseline classifiers (SC_top_), its performance is restricted to the best baseline classifier (kNN). This difference between the SC ensemble strategy variants is attributed to the fact that the SC_all_ variant creates more representative dataset information for the meta-classifier. Furthermore, the SC_all_ ensemble archives competitive MDE valuations (the fewest, the better) with only five misclassified elements, indicating that five sick elements are misclassified as healthy. However, this result can be related to the dataset characteristics, specifically the presence of overlapping attributes where only a small number showed significant variances. In the case of voting ensemble variants (VC_all_ or VC_top_), the VC ensemble strategy is limited to the kNN (the best baseline classifier) independently of using the complete set (all) or the three most promising baseline classifiers.

When only the baseline classifiers are evaluated, the kNN classifier is the most outstanding baseline classifier (using the Euclidean distance metric and one neighbor), achieving above 0.94 in accuracy, recall, precision, and F1-score compared to NB, LR, LDA, QDA, SVM, ANN, and DT baseline classifiers. Interestingly, the second-best baseline classifier is the SVM (with a polynomial kernel of seventh degree), whose classification process is also based on geometrical approaches. Also, it is remarkable that the classifiers whose processes are probabilistic-based (NB, LDA, and QDA) yield the worst outcomes and cannot surpass the 0.81 value in the evaluated metrics, whereas the rest of the classifiers can surpass such a value. This inefficiency of the classifiers based on probabilistic approaches is attributed to the high correlation magnitude between the dataset’s attributes, while the classifiers require statistical independence among the attributes.

Finally, for future work, we propose a comprehensive investigation of the impact of reducing the dataset’s attributes on the classifiers. Additionally, exploring alternative scoring methods beyond those employed in this work is considered. In addition, the innovation of classifiers based on the techniques here-reported that achieved the best outcomes is considered, as well as their comparison with other methods documented in the existing literature.

## Figures and Tables

**Table 1:** Popular distance functions of the kNN classifier.

Name	Distance Function $d(x^{\prime },x)$
Euclidean	$\sqrt{\sum_{j=1}^{n}{\left(x_{j}-{x^{\prime }}_{j}\right)}^{2}}$
Manhattan	$\sum_{j=1}^{n}\left|x_{j}-x_{j}^{\prime }\right|$

**Table 2:** Popular kernel functions of the SVM classifier.

Type of Kernel	Kernel’s Function $k(x,x^{\prime })$
Linear	$\langle x^{T}x^{\prime }\rangle$
Polynomial ^1,2^	${\left(\gamma \langle x^{T}x^{\prime }\rangle +r\right)}^{d},\left\{d\in N|d\geq 2\right\}$
Radial Basis Function (RBF) ^1^	$\exp\left(-\gamma {∥x-x^{\prime }∥}^{2}\right)$
Sigmoid ^1,2^	$\tanh\left(\gamma \langle x^{T}x^{\prime }\rangle +r\right)$

^1^ γ is a weight value. ^2^ *r* is a bias value.

**Table 3:** Popular activation functions of the ANN classifier.

Name	Activation Function
Linear (identity)	f(z)=z
Sigmoid (logistic)	$f\left(z\right)=\frac{e^{z}}{1+e^{z}}$
Hyperbolic (tanh)	$f\left(z\right)=\frac{e^{z}-e^{-z}}{e^{z}+e^{-z}}$
Rectified linear unit (relu)	$f\left(z\right)=\left\{\begin{array}{cc} 0, & \mathrm{if}\;z<0\\ z, & \mathrm{otherwise} \end{array}\right.$

**Table 4:** Baseline classifiers’ tuned hyperparameters.

Classifier	Hyperparameters
kNN	*k*-neighbors: 1
	distance metric: Euclidean
NB	-
LR	solver: newton-cholesky
LDA	solver: *svd*
QDA	-
SVM	kernel: polynomial
	degree: 7
ANN	hidden structure: 6 layers, 6 neurons
	activation function: identity
	learning rate: 0.08
	solver: lbfgs
DT	criterion: gini
	max. depth: 10
	min. samples per leaf: 1
	min. samples to split: 3
	splitter: best

**Table 5:** Baseline classifiers results.

Classifier	Accuracy	Precision	Recall	F1-Score		Confusion Matrix			CCE	MDE	Time (s)
						DH	SL	NO			
kNN	**0.9488**	**0.9520**	**0.9488**	**0.9490**	DH	146	4	0	**427**	**4**	1.27
					SL	6	140	4			
					NO	1	8	141			
NB	0.7444	0.7508	0.7444	0.7324	DH	123	23	4	335	21	0.253
					SL	64	69	17			
					NO	0	7	143			
LR	0.8200	0.8295	0.8200	0.8198	DH	114	35	1	369	7	1.057
					SL	30	114	6			
					NO	3	6	141			
LDA	0.7844	0.8069	0.7844	0.7876	DH	119	31	0	353	5	0.1312
					SL	34	111	5			
					NO	12	15	123			
QDA	0.7866	0.7957	0.7866	0.7868	DH	108	38	4	354	15	0.1112
					SL	33	106	11			
					NO	1	9	140			
SVM	0.8533	0.8720	0.8533	0.8492	DH	138	10	2	384	8	33.86
					SL	41	103	6			
					NO	2	5	143			
ANN	0.8155	0.8249	0.8155	0.8154	DH	113	36	1	367	7	210.84
					SL	32	112	6			
					NO	2	6	142			
DT	0.8288	0.8409	0.8288	0.8299	DH	120	29	1	373	6	69.76
					SL	33	112	5			
					NO	1	8	141			

**Table 6:** Ensemble classifiers results.

Classifier	Accuracy	Precision	Recall	F1-Score		Confusion Matrix			CCE	MDE	Time (s)
						DH	SL	NO			
RF	0.8822	0.8889	0.8822	0.8811	DH	137	12	1	397	6	1.278
					SL	29	116	5			
					NO	0	6	144			
VC_top_	0.9488	0.9520	0.9488	0.9490	DH	146	4	0	427	**4**	0.878
					SL	6	140	4			
					NO	1	8	141			
SC_top_	0.9488	0.9510	0.9488	0.9487	DH	146	4	0	427	6	0.914
					SL	6	138	6			
					NO	1	6	143			
VC_all_	0.9488	0.9520	0.9488	0.9490	DH	146	4	0	427	**4**	1.365
					SL	6	140	4			
					NO	1	8	141			
SC_all_	**0.9511**	**0.9534**	**0.9511**	**0.9509**	DH	146	4	0	**428**	5	1.449
					SL	6	139	5			
					NO	1	6	143			

## Data Availability

Data will be made available on request.
